# Comparison of Physicochemical Properties and Metabolite Profiling Using ^1^H NMR Spectroscopy of Korean Wheat Malt

**DOI:** 10.3390/foods9101436

**Published:** 2020-10-11

**Authors:** Yang Soo Byeon, Dabeen Lee, Young-Shick Hong, Seung-Taik Lim, Sang Sook Kim, Han Sub Kwak

**Affiliations:** 1 Research Group of Food Processing, Korea Food Research Institute, Wanju-gun 55365, Korea; 50024@kfri.re.kr (Y.S.B.); Lee.Da-been@kfri.re.kr (D.L.); 2 Department of Biotechnology, College of Life Sciences and Biotechnology, Korea University, Seoul 02841, Korea; limst@korea.ac.kr; 3 Division of Food and Nutrition, Chonnam National University, Gwangju 61188, Korea; chtiger@jnu.ac.kr

**Keywords:** wheat malt, Korean wheat, malt quality, enzyme activity, metabolites

## Abstract

The objective of this study was to compare the physicochemical, enzymatic, and metabolic properties of two control wheat malts imported from Germany and the US to those of malts made from three Korean wheat varieties: *Triticum* *aestivum* L., var. *Anzunbaengi*, *Jokyung*, and *Keumkang*. The qualities and enzyme activities of the Korean wheat malts were generally similar to those of the control wheat malts. The Korean wheat malts had slightly lower diastatic power and enzyme activities related to saccharification. The analysis of metabolites in the wheat malt samples was performed using ^1^H nuclear magnetic resonance (NMR) metabolomics, which identified 32 metabolites that differed significantly among the samples. Most amino acids and lipids were more abundant in the Korean wheat malts than in the control wheat malts. These differences among malts could influence the quality and flavor of wheat beers. Further brewing studies are necessary to identify the association between beer quality and individual malt metabolites.

## 1. Introduction

Wheat (*Triticum aestivum* L.) is one of the oldest cultivated cereals and is a global staple food with a global production volume of 738 million tons in 2017 [1]. In Korea, wheat is the second most consumed grain (next to rice), and annual wheat consumption has increased to 33 kg per capita in 2018. In Korea, three *T. aestivum* varieties called *Keumkang*, *Jokyung*, and *Anzunbaengi* are mainly cultivated, and several researchers have studied the applications of Korean wheat in the food industry. A comparison of physicochemical properties of domestic and imported wheat shows that the quality variation of the domestic wheats was larger than the imported ones [2]. In terms of commercial wheat flours, some domestic wheat flours had a similar sensory profile and consumer acceptability compared to the imported wheat flours in a pan bread study [3]. In terms of producing a value-added wheat ingredient, the functionality of germinated wheat [4] and immature wheat [5] has been reported. To the authors’ knowledge, no study on the production of wheat malt has yet been reported.

Wheat beer is a popular alcoholic beverage, and especially in the last few years, there has been an increase in the global demand for wheat beer [6]. Wheat malt is used as a base malt for brewing with the replacement of 40–60% of the barley malt [7]. Several studies of wheat malts have reported the impact of the physicochemical characteristics on wheat malt quality. Krahl et al. [8] presented the inverse relation between malting parameters and water-extractable arabinoxylan in wheat, rye, and spelt wheat. Faltermaier et al. [9] analyzed the microstructural transformation of unmalted wheat grains to malt using optical tools, and they observed that the metabolic changes in kernels that were verified by malt-quality parameters. Moreover, an appropriate Kolbach index range of 37.6–42.7% for wheat malts was determined by investigating the relationship between the Kolbach index and other parameters, such as the levels of protein degradation, the malt quality, and the enzyme activities in the wheat malts [10].

Quantitative metabolomics provides comprehensive information about the organic compounds in a wide variety of samples [11]. Bettenhausen et al. [12] established the effect of malt metabolites on the chemical properties of finished beer using the untargeted metabolomics approach. They showed that different chemical compositions due to the distinct metabolites in malt based on the barley genotype and environment could affect the quality and sensory attributes of beer as well as the flavor stability of the final product. Metabolites can be analyzed using ^1^H nuclear magnetic resonance (NMR), which is a widely recognized high-throughput tool for the simultaneous analysis of the metabolites comprising a sample. To date, ^1^H NMR-based metabolomics has proven to be a useful technique for identifying chemical compounds and exploring the associations between holistic metabolite variations. It has the advantages of relative ease of sample preparation and high efficiency in quantifying metabolite levels. In addition, compound identification can be readily verified by a comparison with the literature or public NMR data due to the high level of experimental reproducibility [13]. Various studies have analyzed food and plant samples using ^1^H NMR spectroscopy. Song et al. [14] documented the metabotyping of rice leaves and grains for the identification of those of potential eating quality, and Lee et al. [15] profiled the metabolites of green tea using the NMR metabolomics approach.

The key disadvantage of using Korean domestic wheat is its price, since Korean wheat is approximately three times more expensive than imported wheat. Therefore, the use of Korean wheat in foods such as bread, noodles, and snacks would have a significant adverse influence on the cost of ingredients. However, for alcoholic beverages, the cost of ingredients has a relatively lower influence than it does for the aforementioned food items. To the best of our knowledge, no study has yet investigated the production of wheat malt using Korean wheat kernels. Therefore, the objective of this study is to compare three Korean wheat malts made from domestic wheats with two other commercial wheat malts to present the possibility of the use of Korean wheat malts instead of imported ones, which are widely used in Korean breweries. The physicochemical properties and metabolite profiles were measured to identify the impact that the malt characteristics and other factors have on the final product.

## 2. Materials and Methods

### 2.1. Samples and Physicochemical Characteristics of Wheat Kernel

Three Korean wheat varieties (*T. aestivum* L., var. *Anzunbaengi* (AZ), *Jokyung* (JK), and *Keumkang* (KK)) were used for making the wheat malt in this study. The wheat varieties were harvested in 2017 and purchased from local cooperatives at Jinju-si, Hapcheon-gun, and Yeonggwang-gun, respectively. These areas are the major growing regions for the respective varieties in Korea.

The moisture content, kernel weight, diameter, and hardness were measured using the single kernel characteristic system (SKCS) (SKCS4100, Perten Instruments, Huddinge, Sweden). The SKCS measurement was conducted in triplicate on 300 kernels according to the American Association of Cereal Chemists (AACC) method 55-31 [16]. The results for each single kernel were analyzed with the Crush Curve Analysis Software (BRI Australia Ltd., North Ryde, New South Wales, Australia). The kernel size (perimeter, length, and width) and roundness were determined for 25 wheat kernels with an image acquisition system (Hirox Ltd., Tokyo, Japan). The germinative energy (GE), mean germination time (MGT), and germination index (GI) of the wheat samples were measured according to the official Analytica European Brewing Convention (EBC) methods 3.6.2 and 3.7 [17].

### 2.2. Preparation of Samples and the Malting Process

**Figure 1:**
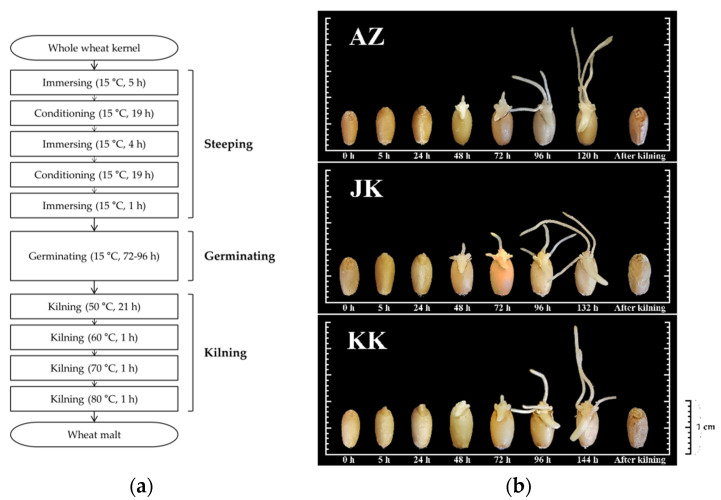
Micromalting procedure (**a**) and representative images of changing wheat kernels during the micromalting (**b**). AZ, JK, and KK represent *Triticum aestivum* L. var. *Anzunbaengi*, *Jokyung*, and *Keumkang*, respectively.

Micromalting was performed according to the method proposed by Mastanjevic et al. [6] with some modifications (Figure 1a). The Korean wheat varieties were evenly distributed in 500 g aliquots into three perforated baskets and incubated in a constant temperature chamber (HK-BI025, Hankuk S&I Co., Hwaseong-si, Korea). The malting procedure was divided into three steps: a steeping stage (48 h), a germination stage (72–96 h), and a kilning stage (24 h). Germination was stopped when the wheat acrospires reached the same lengths as the wheat kernels. Since each Korean wheat variety has different characteristics, the germination time for each sample was different: AZ (120 h), JK (132 h), and KK (144 h) (Figure 1b). The incubator was set at 15 °C and approximately 95% relative humidity during the steeping and germination stages. The kilning process was conducted by an air dryer (HK-DO1000F, Hankuk S&I Co., Hwaseong-si, Korea) for 24 h as follows; 21 h at 50 °C, 1 h at 60 °C, 1 h at 70 °C, and 1 h at 80 °C. After kilning, the rootlets and acrospires of the malted grains were manually removed. The wheat malts were then put into plastic bags and kept in a refrigerator (4 °C) for four weeks to allow for stabilization. Two commercial wheat malts produced in 2017, widely used for wheat beer brewing in Korea, were selected as the control samples that imported from Europe (Weyermann Malting Co., Bamberg, Germany) and the US (Briess Malt & Ingredients Co., Chilton, WI, USA). They were purchased from an online brewery product shopping platform (Hemabrew, Seoul, Korea) in Korea.

The wheat malts were milled using a universal laboratory disc mill (Buhler-Miag GmbH, Braunschweig, Germany) with a 0.2 mm gap between the discs. Congress mashing, a standardized small-scale mashing process used to assess malt quality, was conducted in triplicate according to the EBC guidelines. The malt meals (50 g) and distilled water (200 mL) were put into glass bottles, and the bottles were placed into a water bath (BW-20G, Jeio Tech Co. Ltd., Daejeon, Korea). The samples were stirred frequently using a stainless steel rod during the mashing. The mashing was performed as follows: resting the protein (45 °C, 30 min), increasing the temperature to 70 °C (1 °C/min, 25 min), adding 100 mL hot water (70 °C), and saccharification (70 °C, 60 min). The mash was cooled to room temperature and adjusted to 450 g with distilled water. The mash was then filtered using a filter paper (pore size 8–12 μm; Hyundai Micro Co. Inc., Seoul, Korea) using a glass funnel (Ø 210 mm) in a cold chamber at 4 °C.

### 2.3. Physicochemical Characteristics of Wheat Malt

The malts were analyzed in triplicate according to the official Analytica EBC (2019) to determine their moisture content (%; EBC 2.1), pH (EBC 8.17), wort color (EBC units; EBC 4.7.1), extract yield (%; EBC 4.2), free amino nitrogen contents (mg/100g; spectrophotometry method, EBC 4.10), diastatic power (°WK; EBC 4.12), soluble nitrogen contents (mg/L; EBC 4.3.1), total nitrogen levels (%; EBC 4.9.1), and Kolbach index (%; EBC 4.12). Viscosity was determined at 20 °C using a rotational rheometer (MCR-102, Anton Paar, Graz, Austria) with a PP-50 parallel measuring plate (Ø 50 mm). The viscosity was measured at the 50th measurement point, and the shear stress range at that time was 0.10–0.13 1 s^−1^.

### 2.4. Enzyme Activities of Wheat Malts

The activities of enzymes (α-amylase, β-amylase, and β-endoxylanase) in the wheat malts were measured using the Megazyme assay kits (Megazyme International, Bray, Ireland), according to the manufacturer’s instructions. For α-amylase activity, one unit (U/g) was defined as the amount of enzyme required in the presence of excess thermostable α-glucosidase to release 1 μmol of p-nitrophenol from blocked p-nitrophenyl maltoheptaoside (BPNPG7) in one minute under the defined assay conditions. For β-amylase activity, one unit (U/g) was defined as the amount of enzyme required to release 1 μmol of p-nitrophenol from p-nitrophenyl-β-D-cellotrioside (PNP β-G3) in one minute. The β-endoxylanase activity (U/kg) was measured using xylazyme AX tablets. One unit was defined as the equivalent xylanase activity of the *Trichoderma longibrachiatum* substrate per gram at pH 6.0.

### 2.5. Metabolite Analysis of Wheat Malts

The preparation of the samples for metabolite analysis using a NMR and the processing of the resultant spectra were performed according to the previous study, with some modifications [18]. Each sample was ground to a fine powder using a mortar and pestle with liquid nitrogen, and then freeze-dried for 48 h. A 100 mg sample of the freeze-dried powder was dissolved in 1000deuterium water (D_2_O including 0.0002% trimethylsilylpropanoic acid (TSP), 1000 µL). After this, the mixture was sonicated at room temperature for 20 min, and then centrifuged at 10 °C and 13,000 rpm for 15 min. The supernatants (600 µL) were put into 5 mm NMR tubes. The ^1^H NMR spectrum was acquired using a Bruker Avance 700 spectrometer (Bruker Biospin, Rheinstetten, Germany) equipped with a cryogenic triple-resonance probe and a Bruker automatic injector, and it operated at a frequency of 700.40 MHz ^1^H and at a temperature of 298 K. For the acquisition of 1D NMR spectra, the one-dimensional (1D) nuclear Overhauser effect spectrometry (NOESY) pulse sequence was achieved with water presaturation. For the assignment or identification of the wheat malt metabolite, two dimensional (2D) total correlation spectroscopy (TOCSY) and heteronuclear single quantum correlation (HSQC) NMR experiments were performed (Figure S1). In addition, the assignments were compared with the results published previously [19].

### 2.6. Statistical Analysis

#### 2.6.1. Multivariate Data Analysis for Physicochemical Experiments

An analysis of variance (ANOVA) was conducted using XLSTAT software (Version 2019.4.2., Addinsoft Inc., Paris, France) to determine the differences between the physicochemical properties of the wheat grain and wheat malt samples. When a difference among samples was found, a Fisher’s least significant difference (LSD) multiple comparison was performed to separate the means at *p* < 0.05. A statistical analysis for metabolite differences among the experimental groups was conducted using SPSS statistical software version 21.0 (IBM Co., Chicago, IL, USA). ANOVA and Duncan’s multiple range test were conducted to determine significant differences across the samples in the relative amounts of metabolites. The amounts of individual metabolites in the wheat malt extracts were calculated using the integral area of the peak in the 1D ^1^H NMR spectra corresponding to the metabolite.

#### 2.6.2. Spectral Processing and Multivariate Data Analysis for Metabolites Analysis

All of the 1D NMR spectra data acquired from the wheat malts extracts were manually performed. These spectra were achieved for baseline distortions and correction of the phase using TOPSPIN software (Version 3.2, Bruker Biospin, Rheinstetten, Germany). Subsequently, ASCII-formatted data were analyzed using MATLAB R2010b (Mathworks, Inc., Natick, MA, USA) where the spectra were aligned using the *i*coshift method as described by Savorani et al. [20], and then we conducted multivariate statistical analyses without bucketing or binning. The regions of NMR spectra corresponding to TSP (−0.5–0.5 ppm) and residual water (4.74–4.90 ppm) were discarded to eliminate the effects of imperfect water suppression after the normalization and spectra alignment. A principal component analysis (PCA; an unsupervised pattern recognition method) and orthogonal projections to latent structures-discriminant analysis (OPLS-DA; a supervised pattern recognition method) were conducted using a SIMCA-P (Version 11, Umetrics, Umea, Sweden). The OPLD-DA loading plots were generated using MATLAB (Mathworks, Inc., Natick, MA, USA) to present pairwise comparison between two samples with a script developed from Imperial College London, UK. These OPLS-DA models corresponded to the correlation coefficient between the variables and the experimental classes, and they combined the backtransformed loading of the predictive component with the concentration variables. The variations and discrimination weights between the classes in the OPLS-DA models were expressed by a color code (square of correlation coefficient), as described by Cloarec et al. [21]. The values R^2^X and Q^2^ values explained the quality of the model. The R^2^X is the proportion of variance in the data explained in the model, and the Q^2^ value is the proportion of variance in the data predictable of the model; these two values indicate the goodness of fit of the model and predictability, respectively. Permutation tests were repeated 200 times, which were used together with a 7-fold cross-validation of the OPLS-DA model.

## 3. Results and Discussion

### 3.1. Grain Properties of Korean Wheat Varieties

The physicochemical properties of the three Korean wheat varieties are shown in Table 1. The moisture content of each variety differed significantly across the samples (*p* < 0.05). The ranges of diameter, length, width, and perimeter of the Korean wheat kernels were 2.74–3.02, 6.22–7.01, 3.03–3.41, and 15.14–17.14 mm, respectively, and they were significantly different across the kernels (*p* < 0.05). The diameter, length, width, and perimeter were largest for the JK, followed by the KK and then the AZ. Thousand-kernel weights of the whole wheat grains ranged from 35.39–46.51 g and varied significantly among the three wheat kernels (*p* < 0.05). The AZ wheat kernels had a relatively smaller kernel size and lower mass compared to the other samples used in this study. Thousand-kernel weights of the whole wheat grains ranged from 35.39–46.51 g and were significantly different among the three wheat kernels (*p* < 0.05). The AZ wheat kernel had a relatively smaller kernel size and lower mass property among the samples used in this study.

These results were similar to those of a previous study that reported the kernel size of Korean wheat varieties [22]. The hardness index by the SKCS was significantly lower for the AZ (27.39) than for the JK (64.29) or the KK (57.51). Based on the hardness index, the SKCS categorizes wheat kernels as hard (above 50) or soft (below 50) wheats [23]. The JK and KK can be classified as hard wheats, while the AZ is a soft wheat. The roundness of all samples was not significantly different across varieties.

The germinative capacity for potential malting of the Korean wheat varieties was also assessed (Table 1). The GE results were between 95.75 and 96.83%, and they were not significantly different across the varieties. Frančáková et al. [24] reported that the GE is related to the germination rate; therefore, the wheat varieties in this study would have similar germination rates for malting. Since all the Korean wheat varieties had GE values above 95%, these varieties are all appropriate for malting [17]. The MGT and GI were significantly different across the samples, i.e., 1.15 and 8.72 for the JK compared to 1.09 and 9.20 for the AZ and 1.08 and 9.29 for the KK (*p* < 0.05). The GI value is an indicator of the germination potential; a value close to 10 indicates a high quality and homogeneity of the malt [24]. The results showed that Korean wheat varieties (AZ, JK, and KK) are of good quality for grain dormancy.

### 3.2. Wheat Malt Properties

The physicochemical properties of the control wheat malts from Germany (GW) and the US (UW) and the AZ (KWA), JK (KWJ), and KK (KWK) Korean wheat malt varieties are shown in Table 2. The moisture contents of the five wheat malts were in the range of 4.1–5.2%. Different storage conditions and drying levels at the malt kilning stage account for the differences. The colors of the worts ranged between 3.5 and 5.6 EBC units, which equates to a bright golden color.

The extract yield of a malt, which is an indicator of the fermentable sugar available for generating alcohol, depends on the starch and protein contents of the grains [25]. All of the samples had extract yields around 80%.

The free amino nitrogen in a common wheat malt is 90–120 mg/100g [26]. The free amino nitrogen values of the Korean wheat malts were mostly higher than GW and UW. The KWJ had the highest value (151.6 mg/100g), followed by KWK (136.0 mg/100g), and KWA (124.5 mg/100g). Brewing that uses Korean wheat malts may influence the proliferation of yeasts and the development of off-flavors due to the high free amino nitrogen values [26].

Diastatic power is an indicator of the hydrolysis capacity of the amylolytic enzymes during the mashing process and is estimated by measuring the alpha and beta amylase activities of the malt [17]. The diastatic power values of the GW and UW were 475.8 and 469.3 °WK respectively, which were significantly higher than the values for the Korean wheat malts (*p* < 0.05), i.e., KWA (415.3 °WK), KWJ (400.5 °WK), and KWK (383.0 °WK). All the malts in this study were over the minimum value (200 °WK) for brewing [27].

Wort viscosity is used as an indicator of process efficiency during wort filtration [28]. Generally, a low wort viscosity is preferred for an efficient brewing process, and the recommendation for wheat malt is a viscosity of less than 1.80 mPa·s [26]. The viscosity of the worts differed significantly among samples (*p* < 0.05). The UW variety had the lowest viscosity (1.91 mPa·s), while the KWA had the highest viscosity (2.84 mPa·s). Since wheat or wheat malts are mixed with barley malt in brewing, the high viscosity levels in this study may not influence the filtration issue during mass production.

Soluble nitrogen was significantly higher for the KWJ variety at 1140.0 mg/L, while the other samples ranged from 775.4–848.8 mg/L (*p* < 0.05). The recommended soluble nitrogen content for typical wheat malts is in the range of 650–780 mg/L [26]. These values for the Korean wheats, and particularly the KWJ, are too high for brewing. The total nitrogen content was significantly higher in the Korean wheat malts (2.24–2.42%) than in control wheat malt samples (1.74–1.79%). The Kolbach index, which is determined by the soluble to total nitrogen ratio, reflects the level of proteolysis during the malting and mashing processes [27]. Of the Korean wheat malts, the KWA had the lowest value at 32.9%, while the KWJ had the highest Kolbach index at 47.3%. In fermentation, nitrogen is the most important nutrient for yeast growth, but excessively eluted nitrogen from the malt could be responsible for the formation of undesirable off-flavors in the beer [12,29]. Although the KWA was a malt with a high total nitrogen content, the soluble nitrogen was observed to be much lower. With regard to avoiding off-flavors in wheat beer, the KWA variety might be the most suitable ingredient for brewing among Korean wheat malts. A brewing study with these wheat malts is necessary as a follow-up study.

### 3.3. Enzyme Activities of Wheat Malts

The enzyme activities of wheat malt are shown in Table 3. Wheat kernels contain various types of glucosidase, such as α-amylase, β-amylase, and limit dextrinase [26]. The GW had significantly higher α-amylase activities (*p* < 0.05) than the others, followed by the UW. The three Korean wheat malts had low α-amylase activity (42.04–46.01 U/g). The β-amylase activities ranged between 10.84 and 14.11 U/g, and was not shown that clear trends between the control and Korean wheat malt samples. These amylolytic enzymes were present in the original kernels and were not influenced by the malting process [26]. The β-endoxylanase activities were significantly different not only between the Korean and the commercial malts, but also among the Korean malts (*p* < 0.05). The highest activity was in the UW, at 93.12 U/kg, followed by the KWJ and KWA, at 70.93 and 60.02 U/kg, respectively. Non-starch-degrading enzymes, including glucanase and xylanase, are produced during the germination process and may decrease the wort viscosity [26].

### 3.4. Metabolites Profile from Wheat Malts

**Figure 2:**
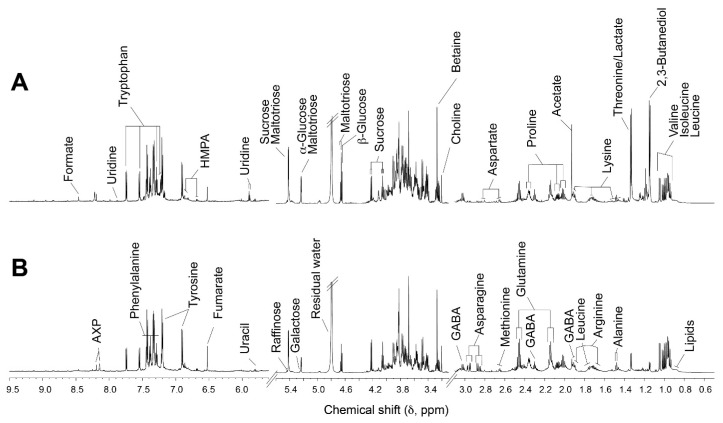
Representative ^1^H 700 MHz nuclear magnetic resonance (NMR) spectra of wheat malt extracts of (**A**) German wheat malt (GW) and (**B**) Korean wheat malt *Keumkang* (KWK) cultivar. HMPA, 4-hydroxy-3-methoxyphenylacetate; AXP, adenosine mono-/di-/tri-phosphate; GABA, gamma-aminobutyrate.

**Figure 3:**
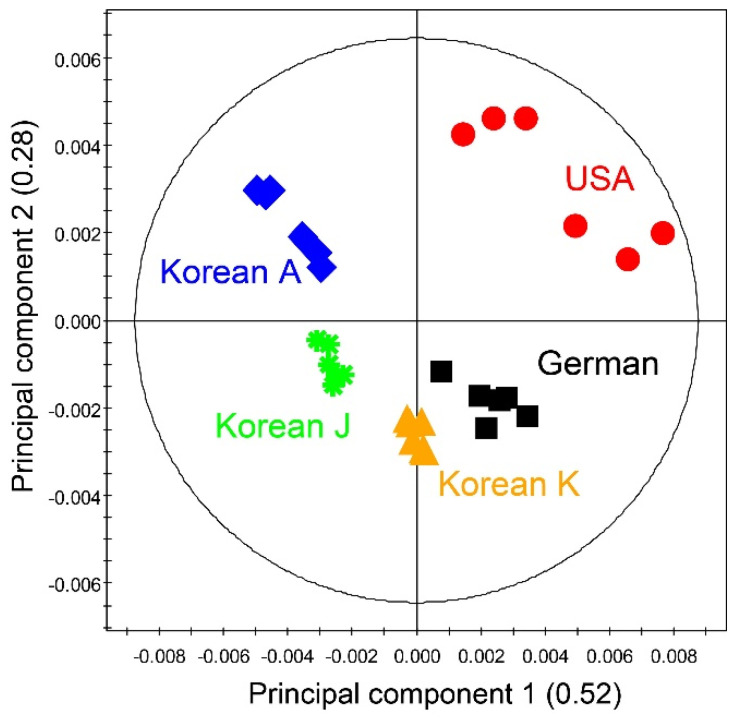
Principal component analysis (PCA) generated from ^1^H NMR spectra of wheat malt extracts according to wheat cultivars and their origins. Korean A, J, and K represents wheat malts from *Triticum aestivum* L. var. *Anzunbaengi*, *Jokyung*, and *Keumkang*, respectively.

The representative ^1^H 700 MHz NMR spectra of wheat malts obtained from GW and KWK are shown in Figure 2. The visualization of the tracked NMR spectra reveals a distinct difference in the chemical compositions of the two samples. The PCA of the metabolites from the wheat malt varieties shows clear metabolic discrimination among the Korean wheat malts, as well as between the Korean and control wheat malts (Figure 3). The first component explained 52% of the total variation, and the second component accounted for 28% of the total variation.

**Figure 4:**
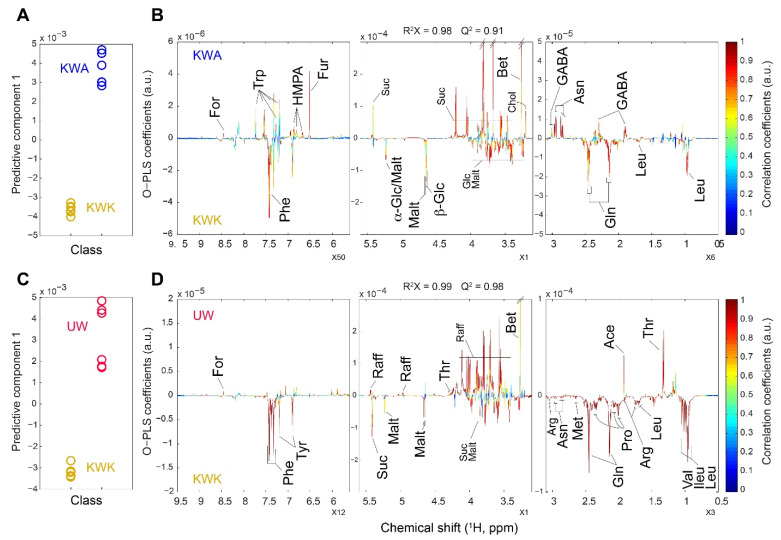
Orthogonal projections to latent structures-discriminant analysis (OPLS-DA) score (**A** and **C**) and loading (**B** and **D**) plots generated from ^1^H NMR spectra of wheat malt extracts with a pairwise plot for metabolic comparisons between KWK and KWA (**A** and **B**) and between KWK and UW (**C** and **D**). GW: German wheat malt, UW: US wheat malt, KWA: Korean wheat malt (var. *Anzunbaengi*), KWJ: Korean wheat malt (var. *Jokyung*), KWK: Korean wheat malt (var. *Keumkang*).

**Figure 5:**
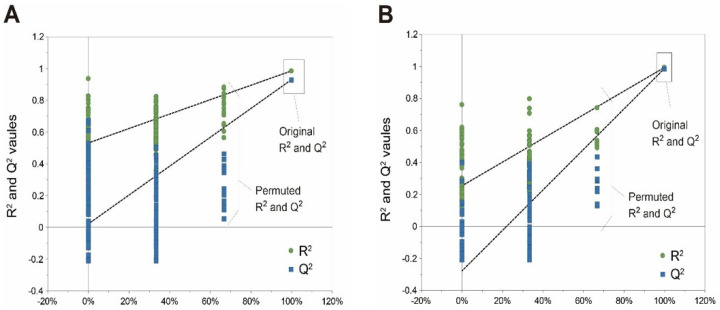
Permutation test in projections to latent structures-discriminant analysis (PLS-DA) models to validate OPLS-DA models. Correlation between original Y-vector and permuted Y-vector: (**A**) between KWA and KWK; (**B**) between KWK and UW given in Figure 3.

The differences between the wheat malts were identified using OPLS-DA modeling, and the OPLS-DA loading plots for the selected wheat malt samples are shown in Figure 4. All OPLS-DA models were generated with one predictive component and one orthogonal component, and they were validated by permutation (Figure 5). The models show good fitness and significant predictability, as indicated by the R^2^X and Q^2^ values respectively: R^2^X = 0.98 and Q^2^ = 0.91 for the model comparing the KWA and the KWK (Figure 4A) and R^2^X = 0.99 and Q^2^ = 0.98 for the model comparing the UW and the KWK (Figure 4C). The KWA shows relatively high amounts of γ-aminobutyric acid (GABA), asparagine, choline, betaine, sucrose, fumarate, 4-hydroxy-3-methoxyphenylacetate (HMPA), tryptophan, and formate, whereas the KWK had relatively high amounts of leucine, glutamine, β-glucose, maltotriose, α-glucose, and phenylalanine (Figure 4B). The discrimination through OPLS-DA modeling between the UW and KWK is presented in Figure 4D. The levels of threonine, acetate, choline, betaine, sucrose, fumarate, HMPA, tryptophan, and formate were relatively higher in the UW than in the KWK. In contrast, the levels of sucrose, maltotriose, and most of the amino acids were higher in the KWK than in the UW.

**Figure 6:**
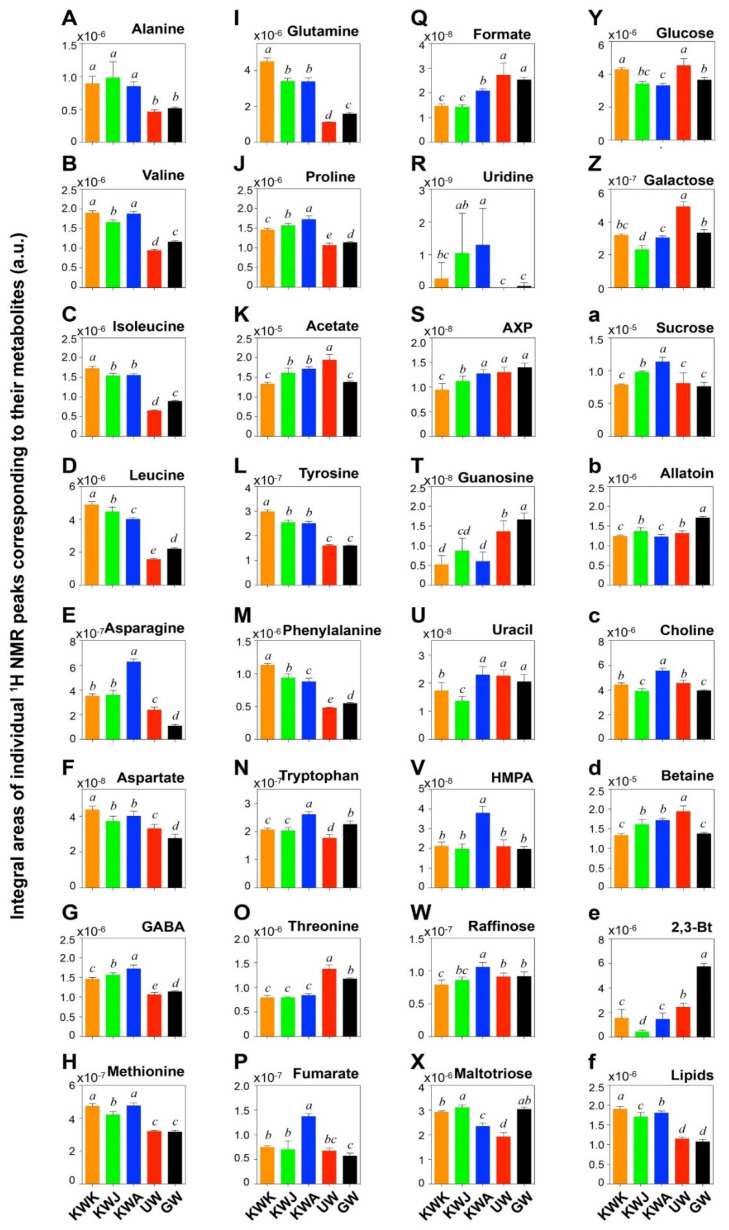
Relative amounts of individual metabolites calculated from integral areas of ^1^H NMR. peaks corresponding the metabolites in extract of various wheat malts and their statistical differences. KWK: Korean wheat malt (var. *Keumkang*), KWJ: Korean wheat malt (var. *Jokyung*), KWA: Korean wheat malt (var. *Anzunbaengi*), UW: US wheat malt, GW: German wheat malt. Different superscripts within the same row represents significantly different at *p* < 0.05 by Duncan’s multiple range test. The ‘a.u.’ is the abbreviation of arbitrary unit.

Overall, 32 metabolites were significantly different among the samples in terms of the relative amounts of individual metabolites (Figure 6). The identified compounds consisted of amino acids (14), organic acids (3), carbohydrates (5), nucleotide metabolites (4), choline metabolites and others (5), and total lipids (1). The key differences in the identifying metabolites among the wheat malts were mainly associated with amino acids. The three Korean wheat malts (KWK, KWJ, and KWA) showed higher levels of certain amino acids compared to the control wheat malts (UW and GW).

Specifically, branched-chain amino acids, such as valine, isoleucine, and leucine, were higher in the Korean malts than in the control ones (Figure 6B–D). These three amino acids have an analogous sequence of biochemical reactions, which are the catabolic (Ehrlich) pathway for the yeast action in the further brewing stage of the fermentation. Oxo acid, which is produced from amino acid via transamination reaction, is converted into aldehyde and then into higher alcohol [29]. Ferreira and Guido [30] reported that the additional valine, isoleucine, and leucine in wort elevated the production of higher alcohols, such as isobutanol, amyl alcohol, and isoamyl alcohol, respectively. These higher alcohols may affect the aroma of the final beer. For example, isobutanol results in a more banana-like aroma, while amyl and isoamyl alcohol impart a solvent-like aroma [31]. Methionine was significantly higher in the Korean wheat malts than in the control wheat malts (*p* < 0.05) (Figure 6H). Methionine is a sulfur-containing amino acid and is directly related to negative flavors such as pungent, rotten eggs and cooked vegetables [32]. Therefore, we can hypothesize that a relatively large presence of a sulfur compound-related substance would have a higher chance in generating unpleasant aroma after brewing. Tyrosine and phenylalanine are precursor amino acids of aromatic alcohols such as tyrosol (bitter) and 2-phenylethanol (floral aroma), respectively [33]. In our tests, Korean wheat malts possess relatively higher amounts of these amino acids (*p* < 0.05) (Figure 6N,M). Although the processing conditions between commercial control wheat malts and Korean wheat malts are different, Korean malts can still be used as an ingredient in brewing. With regard to brewing, differences in types and quantities of metabolites across malts would influence beer quality. The levels of uridine, adenosine mono-/di-/tri- phosphates (AXPs), guanosine, and uracil showed significant differences among the samples (*p* < 0.05) (Figure 6R–U). These are derivatives of degenerated nucleotides or nucleobases as well as products of the metabolic activities, leading to the biosynthesis of purines or pyrimidines during malting [34]. Nucleotide content has a close relationship with malting conditions during steeping, germination, and kilning due to the seed interconversions that occur in higher plant embryos during early germination [19].

Allantoin (5-ureidohydantoin) is a heterocyclic nitrogen secondary metabolite derived from decomposed purines. It is involved in the assimilation and storage of nitrogen in various plants, such as legumes, rice, and wheat, and it can serve as a nitrogen source for germination [35]. Our findings show that allantoin levels were highest in the GW, followed by the KWJ and then the UW (Figure 6b), but no distinctive trend across the samples was observed.

Choline metabolites, such as choline and betaine, are major moieties of membrane phospholipids for the biosynthesis of cell walls in wheat and are also micronutrients [36]. The choline and betaine concentrations differed distinctly among the samples (Figure 6c,d). To the authors’ knowledge, the role of choline metabolites in brewing has not previously been studied.

The Korean wheat malts contained more lipid contents in comparison with two control wheat malts (Figure 6f). Lipids and their oxidized compounds could have a negative effect on beer flavor by converting unsaturated fatty acids to undesirable flavor compounds [37]. Additionally, lipids interact with the protein, which acts on the stability of the gas bubble, resulting in disturbing the foam stability of beer [38]. Therefore, a relatively high lipid content observed in Korean wheat malts may generate adverse effects on the quality of beer flavor and foam stability.

In general, metabolite composition between Korean wheat malts and the imported malts are different, and these differences will generate different flavors when brewed. In the metabolomics study on barley malt and beer, Bettenhausen et al. [12] found that the metabolite profiles of six barley malts from different genotypes and malting locations influenced the chemical and sensory aspects of beer quality. In their study, leucine, methionine, phenylalanine, glutamine, and uridine were positively related to the perceived sensory characteristics of sweet, bread crust, honey, hay, and fruity, respectively. As follows, the metabolite differences across the samples in our study may likewise generate flavor differences when brewed. Additional brewing studies will be necessary to compare how these metabolite differences affect beer properties.

## 4. Conclusions

This is the first study to investigate the physicochemical properties and metabolite profiles of Korean wheat varieties for malt making. The results can demonstrate to Korean small brewers what the physicochemical characteristics of Korean wheat malts are and can deliver them ideas in the use of Korean wheat malt for their brewing. Korean wheat varieties were demonstrated appropriate for malt making, although there are some differences when compared to imported commercial malts. The physicochemical properties of the Korean wheat malts were comparable with control wheat malts in general; the Korean malts differed only in having slightly lower diastatic power and enzyme activities than the imported ones. However, there were several differences in metabolite profiles between samples. The Korean wheat malts were clearly separated from the control samples by the PCA, and most amino acids and lipids were more abundant in the Korean wheat malts than in the imported wheat malts. These differences in metabolite composition between Korean wheat malts imported wheat malts may influence the quality and flavor of the final product, i.e., wheat beer. However, we could not conclude thoroughly the differences that solely came from the different varieties of wheat due to the different processing conditions between imported and Korean wheat malts. In addition, the comparison was conducted using only 2017 crops rather than using crops over several years. Therefore, climate and geological issues were not thoroughly considered in this study. Although some limitations exist in this study, the physicochemical and metabolite properties of Korean wheat malts using three major varieties can present the possibility of wheat malts from Korean wheat varieties. The results of this study could provide initial information regarding the development of malt processing conditions for Korean wheat. The limitations in this study should be considered in further Korean wheat malt study to scale up for mass production.

## Figures and Tables

**Table 1:** Physicochemical and germinative properties of Korean wheat kernels.

	AZ ^1^	JK	KK
Wheat kernel characteristics			
Moisture content (%)	11.50 ± 0.03 ^c^	12.11 ± 0.01 ^b^	12.46 ± 0.02 ^a^
Diameter (mm)	2.74 ± 0.01 ^c^	3.02 ± 0.04 ^a^	2.93 ± 0.01 ^b^
Length (mm)	6.22 ± 0.03	7.01 ± 0.24 ^a^	6.48 ± 0.03 ^b^
Width (mm)	3.03 ± 0.06 ^c^	3.41 ± 0.05 ^a^	3.19 ± 0.03 ^b^
Perimeter (mm)	15.14 ± 0.16 ^c^	17.14 ± 0.48 ^a^	15.95 ± 0.09 ^b^
Thousand-kernel weight (g)	35.39 ± 0.31 ^c^	46.51 ± 1.35 ^a^	41.65 ± 0.52 ^b^
Hardness index	27.39 ± 0.37 ^c^	64.29 ± 1.77 ^a^	57.51 ± 0.64 ^b^
Roundness	1.27 ± 0.01 ^ns^	1.27 ± 0.01	1.27 ± 0.01
Germinative properties			
Germinative energy (%)	96.83 ± 0.76 ^ns^	95.75 ± 0.25	96.08 ± 0.76
Mean germination time (day)	1.09 ± 0.02 ^b^	1.15 ± 0.03 ^a^	1.08 ± 0.02 ^b^
Germination index	9.20 ± 0.12 ^a^	8.72 ± 0.19 ^b^	9.29 ± 0.13 ^a^

^1^ AZ, JK and KK represent *Triticum aestivum* L. var. *Anzunbaengi*, *Jokyung*, and *Keumkang*, respectively. Note: Different superscript letters within the same row represent significantly different at *p* < 0.05 by Fisher’s least significant difference (LSD) test; ns: nonsignificant at *p* < 0.05.

**Table 2:** Malt quality properties of wheat malt.

	Control			Korean Wheat Malt		
	GW ^1^	UW	KWA		KWJ	KWK
Moisture content (%)	4.1 ± 0.3 ^b^	5.1 ± 0.1 ^a^	5.2 ± 0.1 ^a^		4.1 ± 0.5 ^b^	4.3 ± 0.2 ^b^
pH	5.96 ± 0.01 ^d^	6.18 ± 0.02 ^b^	6.24 ± 0.01 ^a^		6.09 ± 0.02 ^c^	6.20 ± 0.02 ^b^
Color (EBC units)	3.7 ± 0.1 ^d^	4.8 ± 0.2 ^b^	3.5 ± 0.2 ^d^		5.6 ± 0.1 ^a^	4.1 ± 0.1 ^c^
Extract (%)	81.9 ± 1.3 ^a^	82.0 ± 0.7 ^a^	80.1 ± 0.7 ^ab^		79.5 ± 1.2 ^b^	80.1 ± 1.5 ^ab^
Free amino nitrogen (mg/100g)	114.8 ± 5.1 ^cd^	106.1 ± 4.9 ^d^	124.5 ± 6.6 ^bc^		151.6 ± 6.6 ^a^	136.0 ± 8.3 ^b^
Diastatic power (°WK)	475.8 ± 3.5 ^a^	469.3 ± 8.7 ^a^	415.3 ± 10.6 ^b^		400.5 ± 11.5 ^bc^	383.0 ± 15.7 ^c^
Viscosity (mPa·s)	2.20 ± 0.09 ^bc^	1.91 ± 0.08 ^d^	2.84 ± 0.21 ^a^		2.00 ± 0.14 ^cd^	2.38 ± 0.08 ^b^
Soluble nitrogen (mg/L)	827.2 ± 26.2 ^bc^	775.4 ± 7.9 ^d^	795.0 ± 8.5 ^cd^		1140.0 ± 54.7 ^a^	848.8 ± 26.2 ^b^
Total nitrogen (%)	1.79 ± 0.01 ^c^	1.74 ± 0.02 ^d^	2.42 ± 0.01 ^a^		2.41 ± 0.01 ^a^	2.24 ± 0.01 ^b^
Kolbach index (%)	46.2 ± 0.4 ^ab^	44.7 ± 0.7 ^b^	32.9 ± 0.4 ^d^		47.3 ± 2.3 ^a^	38.0 ± 1.2 ^c^

^1^ GW: German wheat malt, UW: US wheat malt, KWA: Korean wheat malt (var. *Anzunbaengi*), KWJ: Korean wheat malt (var. *Jokyung*), KWK: Korean wheat malt (var. *Keumkang*). Note: Different superscript letters within the same row represent significantly different at *p* < 0.05 by Fisher’s LSD test.

**Table 3:** Enzyme activities of wheat malts.

	Control			Korean Wheat Malt		
	GW ^1^	UW	KWA		KWJ	KWK
α-Amylase (U/g)	73.67 ± 4.01 ^a^	56.39 ± 2.40 ^b^	46.01 ± 0.78 ^c^		43.63 ± 1.19 ^c^	42.04 ± 0.72 ^c^
β-Amylase (U/g)	12.92 ± 0.07 ^c^	14.11 ± 0.02 ^a^	11.92 ± 0.12 ^d^		10.84 ± 0.06 ^e^	13.30 ± 0.12 ^b^
β-Endoxylanase (U/kg)	50.89 ± 3.93 ^d^	93.12 ± 6.83 ^a^	60.02 ± 2.13 ^c^		70.93 ± 1.33 ^b^	46.38 ± 0.95 ^d^

^1^ GW: German wheat malt, UW: US wheat malt, KWA: Korean wheat malt (var. *Anzunbaengi*), KWJ: Korean wheat malt (var. *Jokyung*), KWK: Korean wheat malt (var. *Keumkang*). Note: Different superscript letters within the same row represent significantly different at *p* < 0.05 by Fisher’s LSD test.

## Supplementary Materials

The following are available online at https://www.mdpi.com/2304-8158/9/10/1436/s1, Figure S1: Typical two-dimensional (2D) spectrum of Korean wheat malt extract.
