# Supplementary material for: Comparison of Physicochemical Properties and Metabolite Profiling Using 1H NMR Spectroscopy of Korean Wheat Malt
Source: Foods. 2020 Oct 11;9(10):1436. doi: 10.3390/foods9101436 (PMC7600480; doi:10.3390/foods9101436)
Supplement: Supplementary file 1 [file foods-09-01436-s001.pdf]

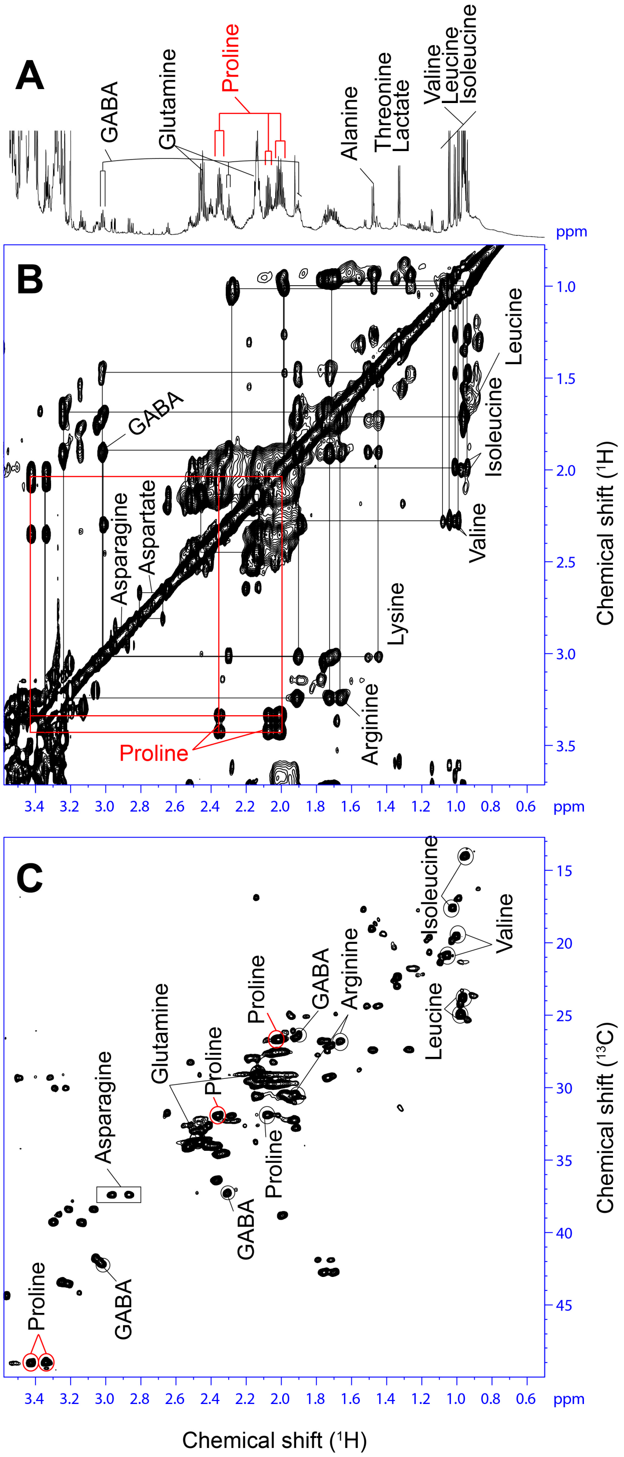

Figure S1. Typical two-dimensional (2D) spectrum of Korean wheat malt extract. Representative one-dimensional (1D) ^{1}H 700 MHz NMR spectra was acquired by using 'NOESY' sequence with water suppression (A). The 2D ^{1}H –^{1}H TOCSY spectrum displays the spin – spin correlation through bonds for each metabolite as cross peaks, symmetrically positioned around the diagonal (B). The 2D ^{1}H – ^{13}C HSQC spectrum displays C – H correlations with carbon chemical shift scale being on the vertical axis (C). Assignment of proline in the wheat malt extract were highlighted with red color as typical metabolite in the current typical 2D NMR experiment.
